# Tritordeum: Promising Cultivars to Improve Health

**DOI:** 10.3390/foods13050661

**Published:** 2024-02-22

**Authors:** Salvatore De Caro, Antonella Venezia, Luigia Di Stasio, Donatella Danzi, Domenico Pignone, Gianfranco Mamone, Giuseppe Iacomino

**Affiliations:** 1 Institute of Food Science, National Research Council (ISA-CNR), 83100 Avellino, Italy; salvatore.decaro@isa.cnr.it (S.D.C.); antovenezia@yahoo.it (A.V.); luigia.distasio@isa.cnr.it (L.D.S.); gianfranco.mamone@isa.cnr.it (G.M.); 2 LILT—Istituto Nazionale Tumori IRCCS, Fondazione G. Pascale, 80131 Napoli, Italy; 3 Institute for Sustainable Plant Protection, National Research Council (IPSP-CNR), 75012 Metaponto, Italy; donatella.danzi@ipsp.cnr.it; 4 Institute of Bioethics for Veterinary and Food, 00054 Fiumicino, Italy; domenico.pignone@cnr.it

**Keywords:** cereal, protein-digested samples, gluten-related disorders, proteomics, Tritordeum

## Abstract

Tritordeum is an amphiploides species resulting from the hybridization between durum wheat (*T. durum*) and wild barley (*H. chilense*). This new cereal is considered a natural crop as it is obtained by traditional breeding techniques. Given its appreciable organoleptic characteristics, agronomic features, presence of interesting components, and good technological properties, Tritordeum is of promising interest for the development of health-oriented foods. In this study, we evaluated two registered Tritordeum cultivars, Bulel and Aucan. *T. durum* (Provenzal) was employed as the positive control. The extracted proteins were digested by gastric/pancreatic proteases, and their biological effects on Caco-2 differentiated on transwell inserts were determined. Changes in cell viability, monolayer permeability, organization of F-actin microfilaments, and ER stress triggered by protein-digested samples (DPs) were inspected. Our results showed that exposure to Provenzal-DPs promptly disrupted the tight junction barrier. Conversely, Aucan-DPs did not enhance monolayer permeability, whereas Bulel-DPs exerted only slight effects. Provental-DPs-induced toxicity was also confirmed by changes in cell viability and by the deep reorganization of the enterocyte cytoskeleton. In contrast, Aucan-DPs and Bulel-DPs did not affect monolayer viability and cytoskeleton structure. Overall, our findings suggest that both Tritordeum cultivars could be potential candidates for mitigating the toxicity of wheat flour.

## 1. Introduction

Cereal-based foods are indispensable constituents of the diet in both developed and developing countries as they are a fundamental source of daily energy, carbohydrates, protein, and fiber [1,2]. Cereals and derived products also include a variety of phytochemicals, and there is a growing interest in the potential health benefits that these substances may offer [3,4,5]. Breeding for end-use quality is one of the most important tasks within cereals [6]. Accordingly, Tritordeum is a recently created grain marketed in the world for human consumption [7]. It has been produced by crossing *Hordeum chilense* (a wild barley species) and *Triticum durum* (durum wheat) [8]. The amphidiploid crop (*H. chilense* × *T. durum*, 2n = 6x = 42, HchHchAABB) has accreditation as a natural species since genetic enhancement has been achieved by traditional breeding and selection practices [9]. This cereal has unique nutritional characteristics [10,11], including higher levels of lutein than common wheat, a higher fraction of dietary fiber [12], and is richer in essential minerals and fructans [11,13]. It can be prospectively considered a valuable alternative to traditional wheat for the production of pasta, bread, and bakery products, combining the nutritional quality of barley with the technological quality of durum wheat [10,14]. Interestingly, Tritordeum products have excellent organoleptic properties [10]. Tritordeum is also an attractive crop agronomically, with yields similar to wheat varieties, high resistance to drought and heat stress, and good resistance to pathogens [15,16]. As a result, this crop is increasingly grown in Europe under both conventional and organic farming systems, and recent studies show that conditions in the southern Mediterranean are ideal for its cultivation [17].

In recent years, research has identified various wheat proteins, including gluten and non-gluten proteins, as contributors to adverse conditions such as wheat allergy, celiac disease, and non-celiac gluten/wheat sensitivity in susceptible individuals. Gluten has gained particular attention due to the increasing number of people identified as susceptible to this protein. Some evidence suggests that diverse grains contain different forms of gliadin peptides, which may have varying toxicities for people with gluten-related disorders [18,19]. Additionally, the gluten-free diet is becoming increasingly popular [20,21]. This popularity is likely due to the positive perception of gluten-free foods as being natural and healthy. Besides, there is a growing interest in ancient grains, which have been shown to have a healthier nutritional profile than modern wheat [22]. Of note, levels of gluten in Tritordeum are lower than those found in common wheat [23].

Several studies have shown that Tritordeum consumption has several beneficial effects, including increased antioxidant activity, reduced inflammation, improved blood sugar control, reduced cholesterol levels, and improved gut health [11]. A recent study investigated subtle differences in the protein composition of two commercial varieties of Tritordeum, with potentially different consequences for immunoreactivity and allergenicity [24]. Additionally, the response of non-celiac gluten-sensitive patients to Tritordeum bread was evaluated by assessing gastrointestinal symptoms, the release of gluten immunogenic peptides, and the composition of the gut microbiota [25], and no obvious differences in gastrointestinal symptoms of gluten-sensitive non-celiac subjects were found between the gluten-free and bread study groups [25]. Yet, it has been reported that short-term ingestion of Tritordeum-based bread does not prompt major changes in the diversity or community composition of the gut microbiota in healthy individuals [26]. Another study evaluated the effects of a 12-week diet of Tritordeum-based foods (bread, baked goods, and pasta) in inflammatory bowel disease (IBD) subjects on gastrointestinal symptoms and intestinal barrier integrity: the Tritordeum-based diet was reported to significantly reduce patients’ symptoms [27]. Different research proposed the possibility of reducing abdominal bloating and improving the psychological status of women with IBD with a diet containing Tritordeum [28]. Yet, investigations in vivo indicate Tritordeum as a cereal characterized by multiple nutraceutical specificities [11].

This study explored the protein composition and biological effects of two Tritordeum varieties, Bulel and Aucan, compared to a standard durum wheat cultivar (*Triticum durum*). The impact of different digested protein (DPs) samples was studied in Caco-2 cells differentiated on permeable supports. Proteomics was used to analyze the gastro-resistant protein component comparatively. This comparison aims to shed light on the distinctive protein properties of Tritordeum varieties and their potential impact on human health.

## 2. Materials and Methods

### 2.1. Materials

Flours of *T. durum* (Provental cv) and Tritordeum—Bulel and Aucan cultivars—were provided by IntiniFood (Putignano, BA, Italy). All reagents and solvents were from Sigma-Aldrich (Milan, Italy).

### 2.2. Protein Extraction

Flour (100 mg) was defatted three times with 1 mL of hexane under continuous stirring (30 min, 22 °C). The suspension was centrifuged (5000× *g*, 15 min), and the solvent was discarded. The defatted material was air-dried overnight. Proteins were extracted from defatted flour with 1 mL of UREA 6M and 20 mM DTT under magnetic stirring for 1 h at 37 °C. After centrifugation at 10,000× *g* for 10 min, the supernatant was collected and desalted with the pre-packed Econo-pack 10-DG column (Bio-Rad, Segrate-Milan, Italy), equilibrated and eluted with 50 mM ammonium bicarbonate. The effluent was monitored by UV absorbance at 280 nm (Ultrospec 160 2100 pro, Amersham Biosciences, Milan, Italy). Lastly, desalted proteins were determined by the Bradford method. Protein samples were stored at −80 °C until use.

### 2.3. In Vitro Simulated Gastrointestinal Digestion

The protein fractions were sequentially digested by gastric and duodenal (pepsin-trypsin-chymotrypsin-carboxypeptidase-elastase), as previously described [29]. The resulting digested protein (DPs) samples were stored at −80 °C until use.

### 2.4. Cell Cultures

The Caco-2 cell line was obtained from the American Type Culture Collection (ATCC, Philadelphia, PA, USA). Cells from passage 20 to 30 were cultured in high-glucose Dulbecco’s Modified Eagle Medium (DMEM) supplemented with 20% heat-inactivated fetal calf serum (FCS), 1% non-essential amino acids (NEAAs), 2 mM L-glutamine, 100 U/mL penicillin, and 100 mg/mL streptomycin in a humidified 5% CO_2_ incubator at 37 °C [30]. Cells were subcultured when they reached 80% confluence, and the medium was replaced every other day.

### 2.5. Effects of DPs on Transepithelial Electrical Resistance (TEER)

The effects of DPs on TEER were compared in Caco-2 cells cultured on permeable supports. After 21 days of differentiation, cells formed a polarized epithelial monolayer that acts as a physical and biochemical barrier to the passage of ions and molecules. This system was utilized as a predictive model of the mucosal barrier due to its inherent ability to spontaneously differentiate into polarized cells with morphological and functional features of enterocytes. Caco-2 cells were seeded on 0.4 μm pore size PET membrane transwell inserts (4.2 cm^2^ growth surface area; BD Falcon, Milan, Italy) at 450,000 cells/cm^2^, and cultured for 21 days before use. To enhance the monolayer’s integrity, we first coated the membrane with bovine collagen type I (Gibco, Thermo Fisher Scientific, Milan, Italy). The differentiation of enterocytes was determined by assessing the activity of alkaline phosphatase using p-nitrophenyl phosphate (Sigma-Aldrich, Milan, Italy) as a substrate. TEER was assessed at room temperature (25 °C) by means of an epithelial voltammeter (Millicell ERS-2, Merck-Millipore, Milan, Italy) fitted with a dual planar electrode. Data were expressed as Ω × cm^2^. Inserts with values lower than 800 Ω × cm^2^ were discarded. Following incubation (0–300 min) in the presence or absence of different DPs, the TEER value for three replicate series was averaged; the resultant values were expressed as the change (%) from the baseline level.

### 2.6. Effects of DPs on Cell Viability

The DPs-induced cytotoxicity on Caco-2 was evaluated by measuring the levels of intracellular ATP [31]. Cells were seeded into 96-well plates at a density of 2 × 10^4^ cells per well and allowed to differentiate for 21 days. After differentiation, cells were exposed to 1.0 mg/mL of DPS for 24 h in a serum-reduced medium (5% FCS). Cell differentiation was preliminarily determined, as reported above. After incubation with specific DPs, samples were processed by using the Vialight Plus Bioluminescence Assay Kit according to the manufacturer’s guidelines (Cambrex Bio Sci Rockland Inc., Rockland, ME, USA). Fifty µL of nucleotide-(ATP) releasing reagent was added to each well, and the plate was incubated for 10 min at room temperature. Subsequently, 100 μL of each resulting lysate was transferred to a luminescence-compatible microplate. Luminescence was acquired with a 1-s integration time using a TopCount-NXTTM luminometer (Packard, Downers Grove, IL, USA). ATP levels in cells were quantified as Relative Light Units (RLUs). The reported results were the mean of three determinations ± SD

### 2.7. Effects of DPs on the Structural Organization of F-Actin

The modifications in the structural organization of F-actin microfilaments resulting from exposition to DPs were examined by a fluorescence microscopy method [19]. Caco-2 cells were differentiated on plastic chamber slides under the above-defined growth conditions and exposed to 1.0 mg/mL DP or PBS for 60 min. Subsequently, the monolayers were treated with 3.75% formaldehyde in phosphate-buffered saline (PBS) for five minutes at room temperature to preserve their morphology. Next, Caco-2 were permeabilized with 0.1% TRITON^®^ X-100 in PBS for 5 min, allowing the phalloidin to penetrate the cell membranes and bind to filamentous actin (F-actin). This was followed by two washes with PBS to remove unbound dye. Finally, F-actin microfilaments were visualized by staining them with 50 µg/mL FITC-labeled phalloidin (Sigma-Aldrich). Cytoskeletal structures labeled with FITC were characterized using fluorescence microscopy with an AxioVert 200 inverted microscope (Zeiss, Jena, Germany). All experiments were performed in triplicate to ensure reproducibility and reduce experimental variability.

### 2.8. Effects of DPs on ER Stress

The endoplasmic reticulum (ER) is subject to various stress conditions that can activate a complex signaling network to restore ER homeostasis. IRE1α, a conserved stress sensor of the unfolded protein response, plays a crucial role in this process. It functions as an endoribonuclease that cuts the mRNA of XBP1, thereby promoting the transcription of genes involved in the ER stress response and protein folding. DPs effect on ER stress was assessed by RT-PCR to recognize XBP1 splicing in differentiated monolayers. The possible splicing was detected using specific primers for human XBP1 that identify both the unspliced (XBP1u) and the spliced (XBP1s) mRNA isoforms [32,33]. RNA was isolated by cell cultures and retro-transcribed to cDNA as described [32]. XBP1s and/or XBP1u amplicons were obtained in a 30 µL PCR reaction containing cDNA and 12 pmol of each primer (5′-TTA CGA GAG AAA ACT CAT GGC C-3′; 5′-GGG TCC AAG TTG TCC AGA ATG C-3′). The cycling settings used were 3 min at 95 °C; 40 cycles of 60 s at 95 °C, annealing for 60 s at 55 °C, and extension for 60 s at 72 °C; and 5 min at 72 °C. Reactions were performed in triplicate, and derived products were detected in 2.5% agarose gel containing ethidium bromide (Sigma Aldrich). After electrophoresis, images were recorded with a digital camera (GelDoc 2000 imaging system; Bio-RAD, Segrate-Milan, Italy). The predicted dimensions of the PCR amplicons were 289 bp for XBP1u and 263 bp for XBP1s.

### 2.9. Proteomic Analysis

The protein sample (100 µg) was resuspended in 100 µL of 62.5 mM Tris-HCl pH 8.5, 2% SDS, containing 20 mM DTT and incubated at 50 °C for 1 h. The reduced sample was alkylated with 55 mM IAA for 1 h in the dark and then made salt-free by Zeba Spin Desalting Columns (Thermo Scientific, San Jose, CA, USA). Digestion was carried out using proteomic grade trypsin (Pierce, Thermo Scientific), at an enzyme-protein ratio 1:20 (*w*/*w*). After incubation at 37 °C for 16 h, the sample was desalted with a C18 spin column (Pierce C18 Spin Column, Thermo Scientific). The digested sample was dissolved in a water solution containing 0.1% (*v*/*v*) formic acid and analyzed by LC-MS/MS system, using a Q Exactive Orbitrap mass spectrometer online with an Ultimate 3000 ultra-high-performance liquid chromatography instrument (Thermo Scientific). The peptide sample was loaded through a 5 mm long, 300 mm i.d. pre-column (Thermo Fisher Scientific, Milan, Italy) and separated by an EASY-Spray™ PepMap C18 column (15 cm × 75 mm i.d.), 3 mm particles, 100 Å pore size (Thermo Scientific). The separation was carried out using 0.1% formic acid (eluent A) and 0.1% formic acid in 80% acetonitrile (eluent B) with the gradient elution at a flow rate of 300 nL/min. Tryptic peptides were separated by allying a linear gradient of 4% 45% of eluent B over 90 min, after 5 min equilibration at 4% B.

The Orbitrap mass spectrometer was operated in data-dependent mode. MS1 spectra were acquired in the positive ionization mode, scanning the range 350–1600 *m*/*z* range with a resolving power up to 70,000 full widths at half maximum (FWHM), an automatic gain control (AGC) target of 10^6^ ions and a maximum ion injection time (IT) of 120 ms. MS/MS fragmentation spectra were obtained at a resolving power of 17,500 FWHM. A dynamic exclusion of 10 s was applied. Ions containing one or more than six charges were excluded from MS/MS fragmentation. Spectra were elaborated using the Xcalibur Software version 3.1 (Thermo Scientific). The resulting MS/MS spectra were searched against the *Triticum* and *Hordeum* UniprotKB dataset downloaded in 2022 (https://uniprot.org (accessed on 6 May 2022)), using the MaxQuant platform (https://maxquant.org (accessed on 6 May 2022)) (version 1.6.2.10). Database search parameters were: trypsin as a proteolytic enzyme and a missed cleavage maximum value of 2; Cys carbamidomethylation, fixed modification; Met oxidation, variable protein modifications; 10 ppm mass tolerance for precursor ion; 0.08 Da ppm mass tolerance for MS/MS fragments. Peptide Spectrum Matches (PSMs) were filtered using the target decoy database approach at 0.01 peptide-level false discovery rate (FDR), corresponding to a 99% confidence score, and validation based on the *q*-value (<1 × 10^−5^).

### 2.10. Statistical Analysis

Differences between groups were determined using one-way analysis of variance (ANOVA) followed by Bonferroni correction. Results were expressed as mean ± SD. Statistical significance was determined using a *p*-value cutoff of 0.05. Data visualization and statistical analysis were performed using GraphPad Prism 9 software (GraphPad, San Diego, CA, USA).

Perseus software version 1.6.5.0, developed by the Max-Planck-Institute of Biochemistry (Martinsried, Germany), was employed to conduct statistical analysis of proteome data. The label-free quantitative (LFQ) protein intensities from the MaxQuant analysis were imported and transformed to a logarithmic scale with base 2. The missing LFQ intensity values (NaN) were replaced using low LFQ intensity values from the normal distribution (width = 0.3, downshift = 1.8). Contaminants and invalid values were removed, and only proteins identified with “sequenced peptide > 1” and “unique peptides > 2” were considered. A permutation-based FDR of 5% was used for truncation of all test results. The fold change visualization was restricted to proteins with a statistically significant probability of abundance change (*p*-value < 0.05).

## 3. Results

### 3.1. Caco-2 Cells

The biological effects of DPs were evaluated in Caco-2 cells cultured on transwell inserts. Once differentiated, the cells arranged themselves as a polarized epithelial monolayer with both the functional and morphological features of enterocytes. Biological effects were detected after incubation with different DPs [34,35]. The optimal incubation conditions with DPs were preliminarily determined in pilot studies.

### 3.2. Effect of DPs on Epithelial Permeability

**Figure 1:**
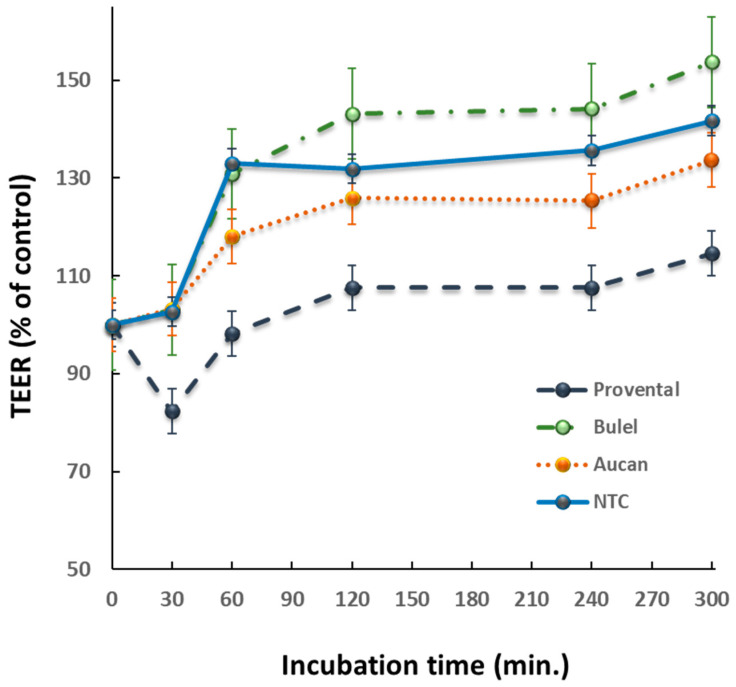
Transepithelial electrical resistance changes were evaluated in differentiated Caco-2 cells following incubation with 1 mg/mL of different DPs. Data were expressed as normalized changes in TEER. Plots are representative of three different experiments. NTC = non-treated control.

Effects of DPs on paracellular permeability were assessed in Caco-2 cells cultured on transwell insert chambers. After 21 days of differentiation, TEER values usually stabilized at >800 Ω × cm^2^. This value was indicative of the integrity of the monolayer and a well-established tight junction-(TJs)-permeability barrier. Exposure to Provental-DPs disrupted the TJs-permeability barrier with a prompt effect detectable after 30 min incubation time. The effects of Tritordeum cultivar DPs on paracellular permeability were comparatively evaluated (Figure 1). TEER values remained almost unchanged when monolayers were incubated with Bulel- or Aucan-DPs. Notably, Bulel-DPs did not increase the permeability of the monolayer, while Aucan-DPs exerted only a slight effect compared with the untreated control. The adverse effect of the positive control (Triticum-DPs) on paracellular permeability was reversible, as TEER values were restored to baseline levels upon the exclusion of DPs from the cell culture medium in agreement with our previous studies [19].

### 3.3. Effects of DPs on Cell Viability

**Figure 2:**
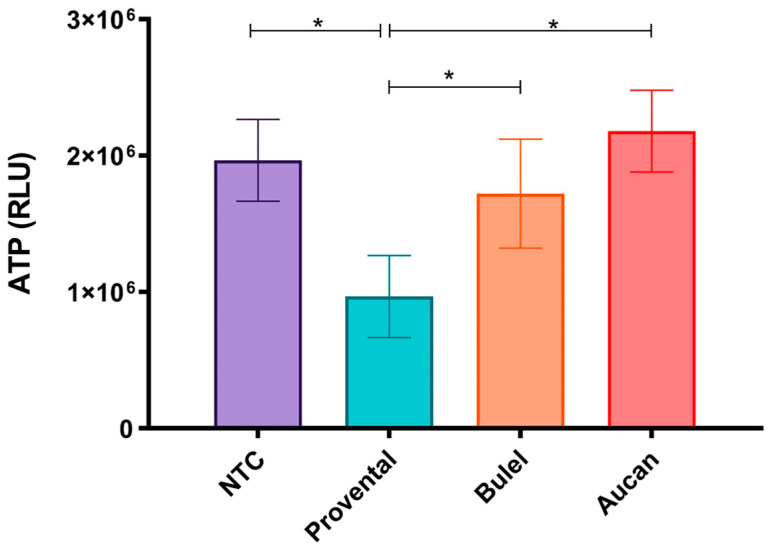
To assess the effects of 1 mg/mL DPs on cell viability, cellular ATP levels were measured in differentiated Caco-2 cells. After 24 h of incubation, luminescence was recorded with a 1-s integration time using a luminometer. Intracellular ATP levels were expressed as relative light units (RLUs). Values are the mean ± SD of three experiments. * *p* < 0.01.

ATP is the key component in the chemistry of every living cell. Its levels are tightly regulated and retained within a fine concentration range. Quantifying the total ATP content in cultured cells is an advantageous approach for determining the number of viable cells, as ATP levels reflect the occurrence of metabolically active cells and, at the same time, represent an easily accessible marker of functional cell integrity [31]. The effects of 24-h incubation to 1.0 mg/mL DPs from different cultivars were determined by monitoring ATP availability in differentiated Caco-2 cells. Exposure to Provental- DPs prompted substantial cytotoxicity documented by a noticeable decline in ATP levels when compared to the untreated control (*p* < 0.05) (Figure 2). In contrast, the viability of cells exposed to both Bulel- and Aucan-DPs remained almost unchanged (*p* > 0.05), thus evidencing the absence of appreciable toxicity exerted by Tritordeum cultivars in the experimental settings. Besides, a reduced ATP availability was observed in Bulel-DPs treated cells as compared to Aucan-DPs, nonetheless, this tendency did not reach a statistical significance (*p* > 0.05). Similar patterns were observed when the monolayers were exposed to DPs for 4 h.

### 3.4. Structural Organization of F-Actin

**Figure 3:**
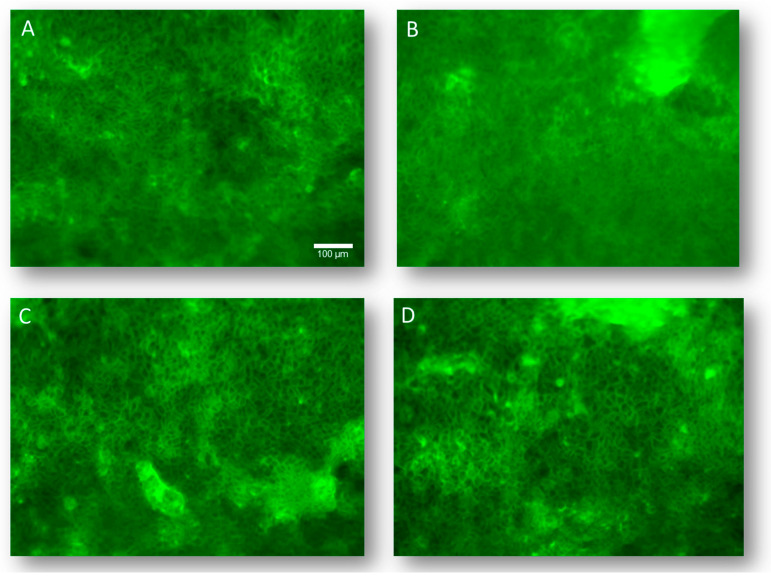
To assess the structural organization of F-actin microfilaments in differentiated Caco-2 cells, cells were grown and differentiated onto chamber slides. After one hour of exposure to 1.0 mg/mL of specific DPs, they were fixed and stained with FITC-labeled phalloidin to visualize microfilament organization under fluorescence microscopy at 200× magnification. Untreated control (**A**); cells incubated with Provental-DPs (**B**); with Bulel-DPs (**C**); with Aucan-DPs (**D**). Images are representative of three experiments performed independently under identical conditions.

In enterocytes and differentiated Caco-2 cells, F-actin filaments form a continuous band in the apical region, close to epithelial junctional complexes (Figure 3A) [36].

This cytoskeletal network is involved in the dynamic modulation of paracellular permeability and intracellular traffic by interacting directly with TJs. Besides, it has been shown that gliadin-derived peptides can alter intestinal permeability by inducing the apical secretion of zonulin, which in turn induces the protein kinase C-mediated polymerization of endo-cellular actin [37,38]. Accordingly, the structural organization of F-actin was addressed in differentiated Caco-2 cells following DPs exposure. Provental-DPs prompted a discernible reorganization of the enterocyte cytoskeleton structure (Figure 3B). Differently, Bulel- and Aucan-DPs did not evoke changes in the cytoskeleton structure (Figure 3C,D) as compared to the untreated control.

### 3.5. Endoplasmic Reticulum Stress

Endoplasmic reticulum (ER) stress is emerging as a common feature in the pathology of numerous diseases.

**Figure 4:**
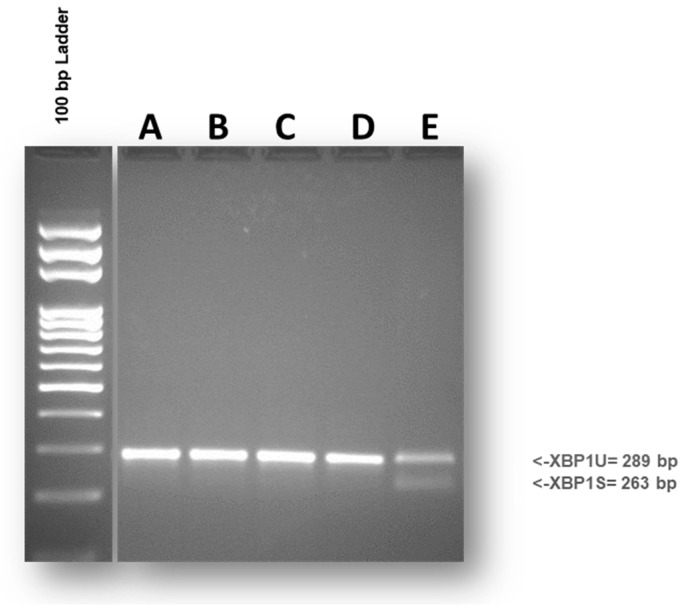
To evaluate the splicing of XBP1 mRNA in 24-h DPs-treated Caco-2 cells, XBP1 was amplified, electrophoresed on a 2.5% agarose gel, and visualized using ethidium bromide staining. The expected sizes of the PCR products were 289 bp for XBP1u and 263 bp for XBP1s. No significant differences were observed between Caco-2 cells exposed to DPs and untreated control. Untreated control (A); cells incubated with Provental-DPs (B); cells incubated with Bulen-DPs (C); cells incubated with Aucan-DPs (D); positive control (E). The gel is representative of three experiments performed independently.

Inositol-requiring transmembrane kinase/endonuclease (IRE1α) serves as a highly conserved stress sensor of the unfolded protein response. Its activity is intricately associated with the stress state of the ER. IRE1α biochemically acts as a ribonuclease that processes the mRNA of the transcription factor X-box binding protein-1 (XBP1). Accordingly, an intron of 26 nucleotides is spliced out from XBP1 mRNA by the activated IRE1α. In most studies, unconventional mRNA splicing can be recognized by RT-PCR performed with primers for XBP1 able to detect both the unspliced (XBP1u) and the spliced (XBP1s) isoforms [33]. In our experimental system, the effects on ER stress from 24-h exposure to different DPs were evaluated. As shown in Figure 4, no differences were recorded between differentiated Caco-2 cells exposed to DPs from Triticum, Tritordeum, and the untreated control. Overall, exposure to various DPs did not evoke XBP1 splicing, thus highlighting that the ER stress response is not a mechanism involved in the cellular response.

### 3.6. Protein Analysis

**Figure 5:**
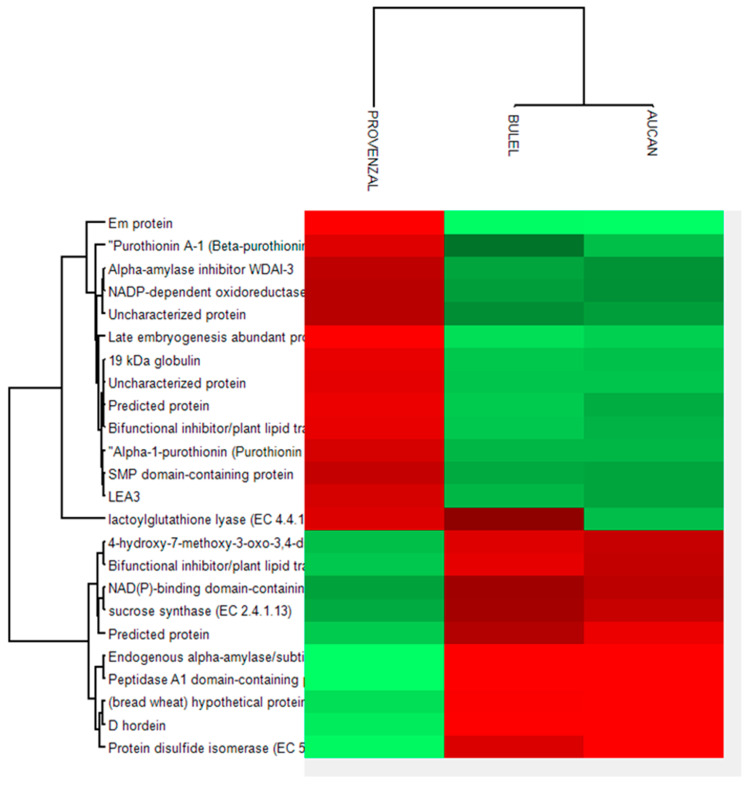
The figure presents the results of the statistical analysis conducted using Perseur software. Twenty-four significantly different proteins were identified between Tritordeum cvs and *Triticum*. The Tritordeum varieties Aucan and Bulel showed comparable protein patterns, including the expression of the lactolylglutathione lyase protein, shared with the Triticum durum species (cv Provental).

The protein composition of Tritordeum DPs cultivars was investigated by LC-MS/MS-based bottom-up proteomics. Overall 596 gene products from *Trititicum* and *Hordeum* species were identified using the MaxQuant search engine. The complete list of gene products identified is reported in Supplementary Table S1. Distinguishing barley-specific proteins from *Triticum* ones was a challenge because of the level of homology between these two species [39]. Most of the identified proteins were involved in the metabolic process. This was not surprising, considering the difficulty of analyzing storage proteins such as gluten or hordein, which requires a targeted analytical approach [40]. Figure 5 shows the result of the statistical analysis performed by Perseus software [41]. A number of 24 significant different proteins between the Tritordeum cvs and *Triticum* were found. The Tritordeum cvs Aucan and Bulel showed similar protein patterns with the expression of protein lactolylglutathione liase, which was common with *Triticum durum* species (Provental cv).

## 4. Discussion

This study aimed to determine the biological effects of Tritordeum versus *Triticum durum*. The effects of DPs were assessed using differentiated Caco-2 cells grown on transwell inserts because differentiated Caco-2 cells form a polarized epithelial monolayer that functions as a physical and biochemical barrier to the movement of ions and molecules. The resulting system was used as a predictive model of human small intestinal mucosa to assess alterations triggered by different DPs. Changes in monolayer barrier function are relevant because antigen trafficking from the apical to the basolateral compartment is a finely controlled process, the dysfunction of which can be reflected in vivo in the delicate balance between induction of immune-inflammatory response and tolerance, with critical repercussions on the onset and progression of gut-related disorders. Of note, when the function of the intestinal epithelial barrier is compromised, it can lead in vivo to a condition known as leaky gut, which allows bacteria, antigens, and toxins to enter the bloodstream. Leaky gut is a very common clinical finding in individuals with conditions such as irritable bowel syndrome, celiac disease, and other disorders.

In our system, the exposure of Caco-2 to Provental-DPs readily disrupted the TJs barrier as predictable. Interestingly, Aucan-DPs did not alter the permeability of the monolayer, whereas Bulel-DPs exerted only minor effects. Our results are in line with a clinical trial of a diet of Tritordeum foods in patients with IBD, in which health benefits appear to occur through an overall improvement in the gastrointestinal barrier [27]. Yet, a recent work compared the in vitro digestibility and immunomodulatory effects of Tritordeum bread prepared with the supplementation of Tritordeum sourdough versus commercial yeast [42]. The authors’ results suggested that Tritordeum bread made with the addition of sourdough or brewer’s yeast showed no significant differences in the composition of the gut microbiota during fecal fermentation in vitro, with the main differences observed in the increase of SCFA concentrations with sourdough Tritordeum bread. Besides, similar to our results, the integrity of the intestinal epithelium, as assessed by the TEER assay, slightly increased after incubation with sourdough Tritordeum bread fermentation.

In 1976, Hudson first described the direct cytotoxic effects of gliadin on immortalized cells [43]. Further research confirmed the effects of growth inhibition and decreased viability induced by digested wheat in Caco-2 cell cultures [44,45]. Remarkably, both Aucan-DPs and Bulel-DPs had no cytotoxic effects on the monolayers, whereas incubation with *T. durum*-DPs caused significant changes in cell viability in our experimental model.

The cytoskeleton is a well-documented target of several toxic agents. Sjolander first characterized the direct damage to the cytoskeleton triggered by the gliadin in cell cultures by analyzing the actin content by immunofluorescence [46]. Interestingly, in our experimental system, Aucan-DPs and Bulel-DPs did not alter cytoskeleton structure, whereas incubation with *T. durum*-DPs, as expected, produced significant toxicity confirmed by an extensive reorganization of the enterocyte cytoskeleton [18,19]. Taken together, our evidence suggests that both cultivars of Tritordeum could be potential candidates for mitigating the intrinsic toxicity of wheat flour.

Unfortunately, the proteomic exploration did not highlight relevant differences between the Tritordeum and *Triticum durum* lines to explain the different toxicity. Notably, the proteomic analysis has mainly identified metabolic proteins, providing, on the contrary, poor information on the gluten content, the analysis of which requires a target analytical approach. Most likely, the different biological behavior is attributable to the gluten content expressed in the two species. Previous studies suggested that Tritordeum cvs have a lower content of indigestible gliadin peptides, which may induce inflammation and intestinal barrier dysfunction [26].

Cereal-based foods have a long history as part of the human diet, dating back far in the mists of time. Numerous studies have confirmed the essential role of whole grains in conferring human health benefits and improving well-being since they contain a wide range of nutrients [47]. Besides, gluten-related disorders have shown increased prevalence in Western countries in recent years, and growing concerns about the possible adverse effects of wheat on a range of diseases in susceptible individuals have been highlighted with the increasing adoption of wheat-free or gluten-free diets [20,21]. However, there is no solid scientific evidence to confirm the health benefits of a gluten-free diet. On the contrary, gluten-free products are usually poor in vitamins, minerals, phytochemicals, proteins, and dietary fiber, all nutrients essential for a well-balanced and healthy diet [48]. In this regard, the need for grain varieties with low toxicity is highlighted [49,50,51]. Of course, given the inter-varieties heterogeneity, both total gliadin content and the proportions of the different gliadin types vary considerably among different wheat cultivars [52].

In this scenario, the prospect of using Tritordeum as a candidate crop with low toxicity and as a source of components for healthful foods is certainly of considerable interest.

## 5. Conclusions

Overall, our results, in agreement with recent scientific literature, suggest that both Tritordeum cultivars analyzed may be potential candidates for mitigating the toxicity of wheat flour, providing further evidence to support their consumption for health purposes and confirming the use of Tritordeum as an attractive opportunity for the food industry to meet today’s consumer demand for healthy, gluten-reduced, and functional products, while also considering the sustainability aspects of this “golden grain.” With this in mind, Tritordeum, while not a safe grain for celiacs, could be useful as a low-toxicity candidate crop and as a source of components for health-valued foods. However, additional studies are required to assess the putative health benefits derived from its consumption in specifically designed intervention studies.

## Data Availability

The original contributions presented in the study are included in the article/supplementary material, further inquiries can be directed to the corresponding author.

## Supplementary Materials

The following supporting information can be downloaded at: https://www.mdpi.com/article/10.3390/foods13050661/s1, Table S1: List of the identified gene products.

## Abbreviations

AGC: automatic gain control; DPs: digested protein samples; ERT: endoplasmic reticulum; FDR: false discovery rate; FITC: fluorescein isothiocyanate; IRE1α: inositol-requiring transmembrane kinase/endonuclease-1; LC-MS: liquid chromatography-mass spectrometry; LFQ: label-free quantitative; NTC: non-treated control; PET: polyester membrane; PSMs: peptide spectrum matches; RLUs: relative light units; TEER: transepithelial electrical resistance; TJs: tight junctions; XBP1: X-box binding protein 1.
